# Supplementary material for: A phase Ib/II clinical study to evaluate the safety and efficacy of topical Arnica tincture to treat non-complicated cutaneous leishmaniasis in Colombia
Source: PLoS Negl Trop Dis. 2025 Aug 18;19(8):e0013123. doi: 10.1371/journal.pntd.0013123 (PMC12373271; doi:10.1371/journal.pntd.0013123)
Supplement: S1 Table — (DOCX) [file pntd.0013123.s001.docx]

**Table S1. Demographic data of enrolled participants**

| **Code** | **Infection region** | **Municipality** | **Street/neighborhood** | **Age** | **Sex** | **Ethnicity** |
| --- | --- | --- | --- | --- | --- | --- |
| PEC02-21_001 | Antioquia | Remedios | Los Ahorcados neighborhood | 28 | F | Mixed |
| PEC02-21_002 | Antioquia | Remedios | Platanales village | 47 | M | Mixed |
| PEC02-21_003 | Antioquia | San Rafael | Las Playas sector | 18 | M | Mixed |
| PEC02-21_004 | Magdalena | Santa Marta | Cañaveral village | 28 | M | Mixed |
| PEC02-21_005 | Antioquia | Valdivia | El Pescado village | 32 | M | Mixed |
| PEC02-21_006 | Antioquia | Buriticá | La Argelina township | 50 | M | Mixed |
| PEC02-21_007 | Boyacá | Puerto Boyacá | El 12 village | 53 | M | Mixed |
| PEC02-21_008 | Chocó | Riosucio | Nueva Esperanza village | 23 | M | Mixed |
| PEC02-21_009 | Antioquia | San Luis | La Josefina village | 18 | M | Mixed |
| PEC02-21_010 | Córdoba | Montería | Moñitos road | 56 | M | Mixed |
| PEC02-21_011 | Chocó | Nuquí | No data | 42 | M | Mixed |
| PEC02-21_012 | Antioquia | Segovia | La Cruzada township | 41 | F | Mixed |
| PEC02-21_013 | Antioquia | Remedios | Urban area | 25 | M | Mixed |
| PEC02-21_014 | Antioquia | Segovia | San Antonio village | 30 | F | Mixed |
| PEC02-21_015 | Antioquia | Frontino | Murrí township | 23 | M | Mixed |
| PEC02-21_016 | Antioquia | Nariño | Guamal village | 41 | M | Mixed |
