# Supplementary material for: A phase Ib/II clinical study to evaluate the safety and efficacy of topical Arnica tincture to treat non-complicated cutaneous leishmaniasis in Colombia
Source: PLoS Negl Trop Dis. 2025 Aug 18;19(8):e0013123. doi: 10.1371/journal.pntd.0013123 (PMC12373271; doi:10.1371/journal.pntd.0013123)
Supplement: S2 Table — (DOCX) [file pntd.0013123.s002.docx]

**Table S2. Medical history of enrolled participants**

| **Code** | **Comorbidity** | **Name** | **Concomitant medication** | **Medication** | **Indication** | **Dosage** | **Frequency** | **Via** | **Start Date** | **End date** | **History of leishmaniasis** |
| --- | --- | --- | --- | --- | --- | --- | --- | --- | --- | --- | --- |
| PEC02-21_001 | No | NA | Yes | Drospirenone + Ethinylestradiol | Contraception | 3 mg + 0.02 mg | Daily | O | APR-2022 | Continuous | No |
| PEC02-21_002 | No | NA | No | NA | NA | NA | NA | NA | NA | NA | No |
| PEC02-21_003 | No | NA | No | NA | NA | NA | NA | NA | NA | NA | No |
| PEC02-21_004 | No | NA | No | NA | NA | NA | NA | NA | NA | NA | No |
| PEC02-21_005 | No | NA | No | NA | NA | NA | NA | NA | NA | NA | No |
| PEC02-21_006 | No | NA | No | NA | NA | NA | NA | NA | NA | NA | No |
| PEC02-21_007 | No | NA | No | NA | NA | NA | NA | NA | NA | NA | No |
| PEC02-21_008 | No | NA | No | NA | NA | NA | NA | NA | NA | NA | No |
| PEC02-21_009 | Yes | Depressive disorder | No | NA | NA | NA | NA | NA | NA | NA | No |
| PEC02-21_010 | No | NA | No | NA | NA | NA | NA | NA | NA | NA | No |
| PEC02-21_011 | No | NA | No | NA | NA | NA | NA | NA | NA | NA | No |
| PEC02-21_012 | No | NA | No | NA | NA | NA | NA | NA | NA | NA | No |
| PEC02-21_013 | No | NA | No | NA | NA | NA | NA | NA | NA | NA | No |
| PEC02-21_014 | No | NA | Yes | Medroxyprogesterone acetate + Estradiol cypionate | Anticonception | 25 mg + 5 mg | Monthly | IM | ENE-2023 | Continuous | No |
| PEC02-21_015 | No | NA | No | NA | NA | NA | NA | NA | NA | NA | No |
| PEC02-21_016 | No | NA | No | NA | NA | NA | NA | NA | NA | NA | No |
