# Supplementary material for: A phase Ib/II clinical study to evaluate the safety and efficacy of topical Arnica tincture to treat non-complicated cutaneous leishmaniasis in Colombia
Source: PLoS Negl Trop Dis. 2025 Aug 18;19(8):e0013123. doi: 10.1371/journal.pntd.0013123 (PMC12373271; doi:10.1371/journal.pntd.0013123)
Supplement: S3 Table — (DOCX) [file pntd.0013123.s003.docx]

**Table S3. Evolution of lesions in each participant during the study**

| **Code** | **Lesion type** | **N° lesion** | **AT scheme^a^** | **Lesion area (mm^2^)** | | | | | | **Outcome** |
| --- | --- | --- | --- | --- | --- | --- | --- | --- | --- | --- |
|  |  |  |  | **D1** | **D30 or D45** | **PTD45** | **PTD60^b^** | **PTD90** | **PTD180** |  |
| PEC02-21_001 | Ulcer | 1 | 45 | 126.83 | 114.62 | 0 | - | 0 | 0 | Definitive Cure |
| PEC02-21_002 | Ulcer | 1 | 45 | 72.48 | NA^c^ | NA | NA | NA | NA | Withdrawal^d^ |
| PEC02-21_003 | Ulcer | 1 | 45 | 160.34 | 161.35 | 0 | - | 0 | 0 | Definitive Cure |
| PEC02-21_004 | Ulcer | 1 | 30 | 158.26 | 96.51 | 0 | - | 0 | 0 | Definitive cure |
| PEC02-21_005 | Ulcer | 1 | 30 | 612.16 | 243.94 | 0 | - | 0 | 0 | Definitive cure |
| PEC02-21_006 | Ulcer | 1 | 30 | 122.47 | 235.65 | 37.66 | - | 0 | 0 | Definitive cure |
|  | Ulcer | 2 |  | 29.69 | 523.70 | 274.80 | - | 0 | 0 | Definitive cure |
| PEC02-21_007 | Ulcer | 1 | 30 | 217.36 | 378.37 | 231.47 | 52,40 | 0 | 0 | Definitive cure |
| PEC02-21_008 | Ulcer | 1 | 45 | 239.18 | 0,00 | 0 | - | ND^e^ | ND | Cure |
| PECO2-21_009 | Ulcer | 1 | 45 | 206.06 | NA | NA | NA | NA | NA | Withdrawal |
| PEC02-21_010 | Ulcer | 1 | 30 | 470.02 | NA | NA | NA | NA | NA | Withdrawal |
| PEC02-21_011 | Ulcer | 1 | 45 | 995.24 | 802.49 | 268.13 | - | 0 | 0 | Definitive cure |
| PEC02-21_012 | Ulcer | 1 | 30 | 27.55 | 36.10 | NA | NA | NA | NA | Withdrawal |
| PEC02-21_013 | Ulcer | 1 | 45 | 447.28 | 0 | 0 | - | 0 | 0 | Definitive cure |
| PEC02-21_014 | Ulcer | 1 | 30 | 168.79 | 271.13 | 0 | - | 0 | 0 | Definitive cure |
| PEC02-21_015 | Ulcer | 1 | 30 | 228.55 | 100.60 | 0 | - | 0 | 0 | Definitive cure |
| PEC02-21_016 | Ulcer | 1 | 45 | 15.57 | 2.36 | 0 | - | 0 | 0 | Definitive cure |

^a^ AT: Arnica Tincture. ^b^Optional visit. ^c^NA: Not applicable. ^d^Participants retired the informed consent or they were retired because they were also treated with glucantime or miltefosine. ^e^Non-compliance in follow-up visits.
