# Supplementary material for: A phase Ib/II clinical study to evaluate the safety and efficacy of topical Arnica tincture to treat non-complicated cutaneous leishmaniasis in Colombia
Source: PLoS Negl Trop Dis. 2025 Aug 18;19(8):e0013123. doi: 10.1371/journal.pntd.0013123 (PMC12373271; doi:10.1371/journal.pntd.0013123)
Supplement: S4 Table — (DOCX) [file pntd.0013123.s004.docx]

**Table S4. Expected Adverse events in enrolled participants during treatment**

| **Code** | **Erythema** | | | | **Burning** | | | | **Pain** | | | |
| --- | --- | --- | --- | --- | --- | --- | --- | --- | --- | --- | --- | --- |
|  | **Presence** | **Intensity** | **Grade** | **Frequency** | **Presence** | **Intensity** | **Grade** | **Frequency** | **Presence** | **Intensity** | **Grade** | **Frequency** |
| PEC02-21_001 | Yes | Mild | 1 | Intermittent | Yes | Mild | 1 | Intermittent | Yes | Mild | 1 | Intermittent |
| PEC02-21_002 | NA | NA | NA | NA | NA | NA | NA | NA | NA | NA | NA | NA |
| PEC02-21_003 | Yes | Moderate | 2 | Once | Yes | Mild | 1 | Once | No | NA | NA | NA |
| PEC02-21_004 | Yes | Mild | 1 | Once | Yes | Mild | 1 | Intermittent | No | NA | NA | NA |
| PEC02-21_005 | Yes | Severe | 3 | Intermittent | Yes | Moderate | 2 | Once | Yes | Mild | 1 | Intermittent |
| PEC02-21_006 | Yes | Mild | 1 | Once | Yes | Mild | 1 | Intermittent | Yes | Mild | 1 | Intermittent |
| PEC02-21_007 | Yes | Mild | 1 | Once | Yes | Mild | 1 | Intermittent | No | NA | NA | NA |
| PEC02-21_008 | Yes | Mild | 3 | Once | Yes | Mild | 1 | Intermittent | No | NA | NA | NA |
| PEC02-21_009 | NA | NA | NA | NA | NA | NA | NA | NA | NA | NA | NA | NA |
| PEC02-21_010 | NA | NA | NA | NA | NA | NA | NA | NA | NA | NA | NA | NA |
| PEC02-21_011 | Yes | Moderate | 2 | Intermittent | Yes | Severe | 3 | Intermittent | Yes | Mild | 1 | Intermittent |
| PEC02-21_012 | NA | NA | NA | NA | Yes | Mild | 1 | Once | No | NA | NA | NA |
| PEC02-21_013 | No | NA | NA | NA | Yes | Mild | 1 | Intermittent | No | NA | NA | NA |
| PEC02-21_014 | Yes | Mild | 1 | Once | Yes | Mild | 1 | Intermittent | Yes | Mild | 1 | Intermittent |
| PEC02-21_015 | No | NA | NA | NA | Yes | Mild | 1 | Once | No | NA | NA | NA |
| PEC02-21_016 | No | NA | NA | NA | Yes | Mild | 1 | Once | No | NA | NA | NA |

**Table S4. Expected Adverse events in enrolled participants during treatment (continuation)**

| **Code** | **Pruritus** | | | | **Edema** | | | |
| --- | --- | --- | --- | --- | --- | --- | --- | --- |
|  | **Presence** | **Intensity** | **Grade** | **Frequency** | **Precence** | **Intensity** | **Grade** | **Frequency** |
| PEC02-21_001 | Yes | Mild | 1 | Intermittent | No | NA | NA | NA |
| PEC02-21_002 | NA | NA | NA | NA | NA | NA | NA | NA |
| PEC02-21_003 | Yes | Moderate | 2 | Intermittent | Yes | Mild | 1 | Intermittent |
| PEC02-21_004 | No | NA | NA | NA | Yes | Mild | 1 | Once |
| PEC02-21_005 | Yes | Severe | 2 | Intermittent | Yes | Severe | 2 | Intermittent |
| PEC02-21_006 | Yes | Mild | 1 | Once | Yes | Mild | 1 | Once |
| PEC02-21_007 | Yes | Mild | 1 | Intermittent | No | NA | NA | NA |
| PEC02-21_008 | No | NA | NA | NA | Yes | Mild | 1 | Once |
| PEC02-21_009 | NA | NA | NA | NA | NA | NA | NA | NA |
| PEC02-21_010 | NA | NA | NA | NA | NA | NA | NA | NA |
| PEC02-21_011 | Yes | Moderate | 2 | Intermittent | No | NA | NA | NA |
| PEC02-21_012 | NA | NA | NA | NA | No | NA | NA | NA |
| PEC02-21_013 | No | NA | NA | NA | No | NA | NA | NA |
| PEC02-21_014 | Yes | Mild | 1 | Intermittent | Yes | Mild | 1 | Intermittent |
| PEC02-21_015 | No | NA | NA | NA | No | NA | NA | NA |
| PEC02-21_016 | No | NA | NA | NA | No | NA | NA | NA |
