# Supplementary material for: A phase Ib/II clinical study to evaluate the safety and efficacy of topical Arnica tincture to treat non-complicated cutaneous leishmaniasis in Colombia
Source: PLoS Negl Trop Dis. 2025 Aug 18;19(8):e0013123. doi: 10.1371/journal.pntd.0013123 (PMC12373271; doi:10.1371/journal.pntd.0013123)
Supplement: S5 Table — (DOCX) [file pntd.0013123.s005.docx]

**Table S5. Other adverse events in enrolled participants during the follow up**

| **Code** | **PTD45** | | | | | | | | | **PTD90** | **PTD180** |
| --- | --- | --- | --- | --- | --- | --- | --- | --- | --- | --- | --- |
|  | **Presence** | **Clinical manifestation # 1** | **Intensity** | **Grade** | **Frequency** | **Clinical manifestation # 2** | **Intensity** | **Grade** | **Frequency** | **Presence** | **Presence** |
| PEC02-21_001 | No | NA | NA | NA | NA | NA | NA | NA | NA | No | No |
| PEC02-21_002 | NA | NA | NA | NA | NA | NA | NA | NA | NA | NA | NA |
| PEC02-21_003 | No | NA | NA | NA | NA | NA | NA | NA | NA | No | No |
| PEC02-21_004 | No | NA | NA | NA | NA | NA | NA | NA | NA | No | No |
| PEC02-21_005 | Yes | Contact dermatitis with bacterial superinfection | Moderate | 2 | Once | Cellulite | Moderate | 2 | Once | No | No |
| PEC02-21_006 | No | NA | NA | NA | NA | NA | NA | NA | NA | No | No |
| PEC02-21_007 | No | NA | NA | NA | NA | NA | NA | NA | NA | No | No |
| PEC02-21_008 | No | NA | NA | NA | NA | NA | NA | NA | NA | NA | NA |
| PEC02-21_009 | NA | NA | NA | NA | NA | NA | NA | NA | NA | NA | NA |
| PEC02-21_010 | NA | NA | NA | NA | NA | NA | NA | NA | NA | NA | NA |
| PEC02-21_011 | No | NA | NA | NA | NA | NA | NA | NA | NA | No | No |
| PEC02-21_012 | NA | NA | NA | NA | NA | NA | NA | NA | NA | NA | NA |
| PEC02-21_013 | No | NA | NA | NA | NA | NA | NA | NA | NA | No | No |
| PEC02-21_014 | No | NA | NA | NA | NA | NA | NA | NA | NA | No | No |
| PEC02-21_015 | No | NA | NA | NA | NA | NA | NA | NA | NA | No | No |
| PEC02-21_016 | No | NA | NA | NA | NA | NA | NA | NA | NA | No | No |
