# Supplementary material for: A phase Ib/II clinical study to evaluate the safety and efficacy of topical Arnica tincture to treat non-complicated cutaneous leishmaniasis in Colombia
Source: PLoS Negl Trop Dis. 2025 Aug 18;19(8):e0013123. doi: 10.1371/journal.pntd.0013123 (PMC12373271; doi:10.1371/journal.pntd.0013123)
Supplement: S6 Table — (DOCX) [file pntd.0013123.s006.docx]

**Table S6. Serum Metabolite Levels (AST, ALT, and Creatinine) Before and After Treatment in Study Participants**

| **Participant ID** | **Screening** | | | | | | **End of the treatment** | | | | | |
| --- | --- | --- | --- | --- | --- | --- | --- | --- | --- | --- | --- | --- |
|  | **AST (U/L)** | **Reference value (U/L)** | **ALT (U/L)** | **Reference value (U/L)** | **Creatinine (mg/dl)** | **Reference value mg/dL** | **AST (U/L)** | **Reference value (U/L)** | **ALT (U/L)** | **Reference value (U/L)** | **Creatinine (mg/dl)** | **Reference value mg/dL** |
| PEC02-21_001 | 19 | 0 - 34 | 27 | 0 - 55 | 0.62 | 0.55 – 1.02 | 14 | 0 - 34 | 10 | 0 - 55 | 0.54 | 0.55 – 1.02 |
| PEC02-21_002 | 21 | 5 - 34 | 22 | 0 - 55 | 0.86 | 0,73 -1.18 | NA | NA | NA | NA | NA | NA |
| PEC02-21_003 | 13 | 14 - 35 | 12 | 8 - 22 | 0.78 | 0.73 -1.18 | 17.71 | 14 - 35 | 14,1 | 8 - 22 | 0.89 | 0.73 -1.18 |
| PEC02-21_004 | 18 | 5 - 34 | 22 | 0 - 55 | 0.8 | 0,73 -1.18 | 25 | 5 - 34 | 39 | 0 - 55 | 0.77 | 0.73 -1.18 |
| PEC02-21_005 | 22 | 5 - 34 | 19 | 0 - 55 | 0.91 | 0.55 – 1.02 | 22 | 11 -34 | 16 | 0 - 45 | 0.88 | 0.73 -1.18 |
| PEC02-21_006 | 32 | 11 - 34 | 64 | 0 - 45 | 0.74 | 0.73 -1.18 | 36 | 11 -34 | 64 | 0 - 45 | 1.17 | 0.73 -1.18 |
| PEC02-21_007 | 30 | 11 - 34 | 62 | 0 - 45 | 0.96 | 0.73 -1.18 | 20 | 11 -34 | 38 | 0 - 45 | 0.96 | 0.73 -1.18 |
| PEC02-21_008 | 22 | 11 - 34 | 18 | 0 - 45 | 0.89 | 0.73 -1.18 | 22 | 11 -34 | 18 | 0 - 45 | 0.94 | 0.73 -1.18 |
| PEC02-21_009 | 13 | 14 - 35 | 7 | 8 - 22 | 1.12 | 0.73 -1.18 | NA | NA | NA | NA | NA | NA |
| PEC02-21_010 | 19 | 11 - 34 | 18 | 0 - 45 | 1.03 | 0.73 -1.18 | NA | NA | NA | NA | NA | NA |
| PEC02-21_011 | 31 | 11 - 34 | 44 | 0 - 45 | 0.88 | 0.73 -1.18 | 24 | 11 - 34 | 30 | 0 - 45 | 0.91 | 0.73 -1.18 |
| PEC02-21_012 | 14 | 11 - 34 | 9 | 0 - 34 | 0.72 | 0.55 – 1.02 | 19 | 11 - 34 | 17 | 0 - 34 | 0.69 | 0.55 – 1.02 |
| PEC02-21_013 | 25 | 14 - 35 | 31 | 9 - 24 | 0.93 | 0.73 -1.18 | 31 | 11 - 34 | 28 | 0 - 45 | 0.85 | 0.73 -1.18 |
| PEC02-21_014 | 19 | 11 - 34 | 14 | 0 - 34 | 0.73 | 0.55 – 1.02 | 18 | 11 - 34 | 14 | 0 - 34 | 0.80 | 0.55 – 1.02 |
| PEC02-21_015 | 27 | 11 - 34 | 41 | 0 - 45 | 0.84 | 0.73 -1.18 | 26 | 11 - 34 | 36 | 0 - 45 | 0.76 | 0.73 -1.18 |
| PEC02-21_016 | 19 | 11 - 34 | 12 | 0 - 45 | 1.25 | 0.73 – 1.18 | 18 | 11 - 34 | 15 | 0 - 45 | 1.18 | 0.73 – 1.18 |

AST: Aspartate aminotransferase; ALT: Alanine aminotransferase. Reference values are included for comparison. “NA” indicates values not applicable or not measured for that participant. All metabolite levels are presented in U/L (AST, ALT) or mg/dL (Creatinine).
