# Supplementary figures and images for: A phase Ib/II clinical study to evaluate the safety and efficacy of topical Arnica tincture to treat non-complicated cutaneous leishmaniasis in Colombia
Source: PLoS Negl Trop Dis. 2025 Aug 18;19(8):e0013123. doi: 10.1371/journal.pntd.0013123 (PMC12373271; doi:10.1371/journal.pntd.0013123)

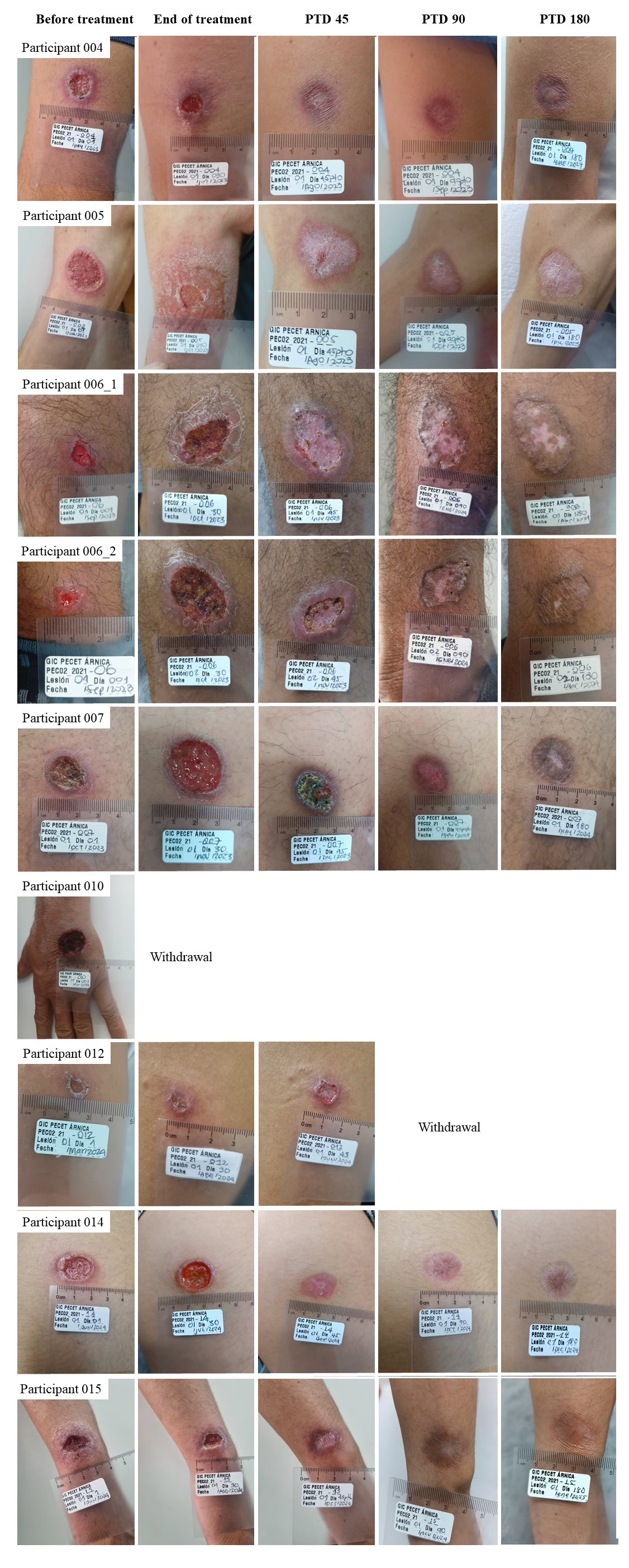

Supplement: S1 Fig — The figures represent individual lesion trajectories across different subjects. D1 indicates the start of treatment, while TD30 denote 30 days of treatment duration, respectively. PTD45, PTD90, and PTD180 correspond to 45, 90, and 180 days post-treatment, respectively. Each line represents the progression of lesion size for an individual participant. (TIF) [file pntd.0013123.s007.tif]

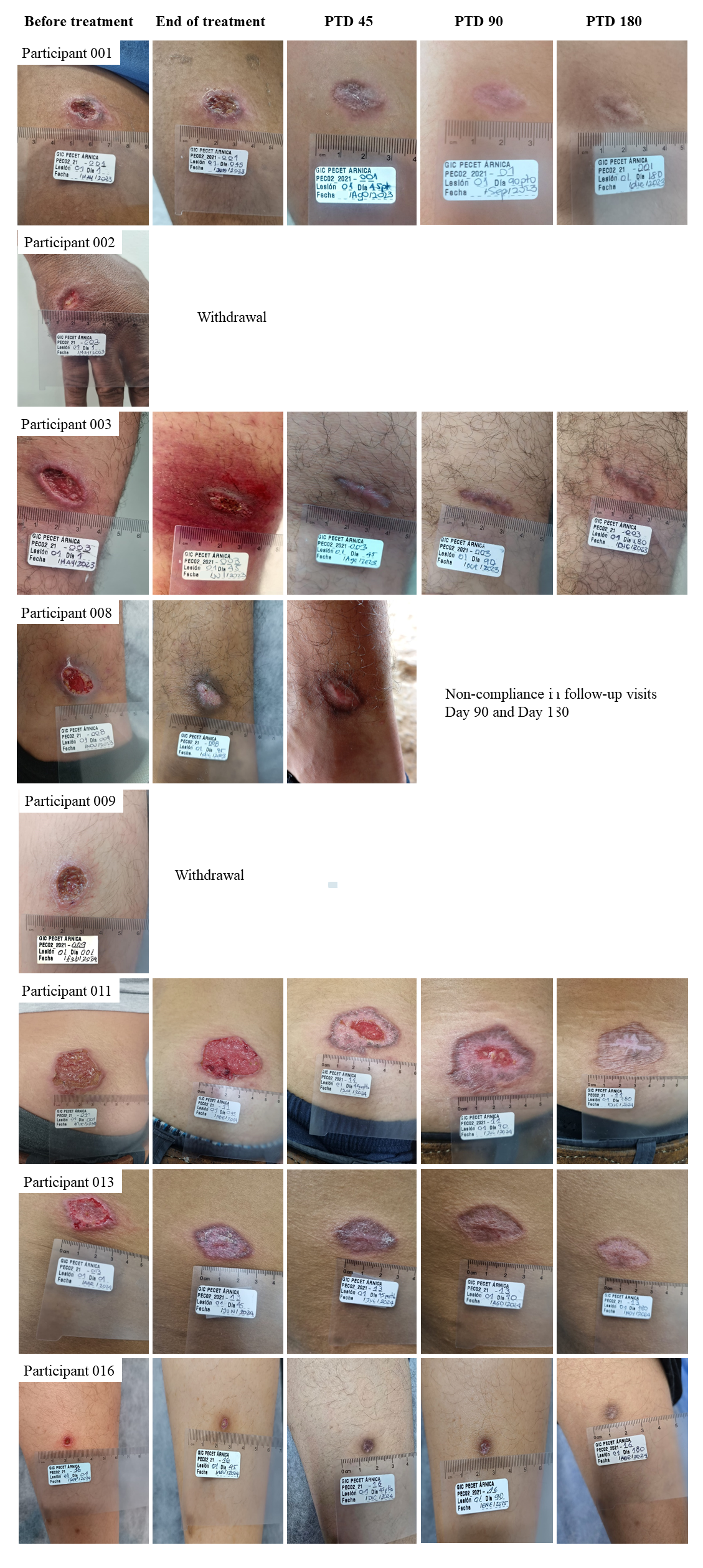

Supplement: S2 Fig — The figures represent individual lesion trajectories across different subjects. D1 indicates the start of treatment, while TD45 denote 45 days of treatment duration, respectively. PTD45, PTD90, and PTD180 correspond to 45, 90, and 180 days post-treatment, respectively. Each line represents the progression of lesion size for an individual participant. (TIF) [file pntd.0013123.s008.tif]
